# Correlation between PLT, MPV, PDW and liver fibrosis and inflammatory activity in patients with NAFLD: A retrospective case-control study

**DOI:** 10.1097/MD.0000000000043815

**Published:** 2025-08-15

**Authors:** Zefeng Zhang, Jun Wang

**Affiliations:** a Wuxi Medical College, Jiangnan University, Wuxi, China; b Department of Clinical Laboratory, Wuxi Fifth People’s Hospital Affiliated to Jiangnan University, Wuxi, China.

**Keywords:** biomarkers, inflammatory activity, liver fibrosis, mean platelet volume (MPV), nonalcoholic fatty liver disease (NAFLD), platelet count (PLT), platelet distribution width (PDW)

## Abstract

This study investigates the association of platelet count (PLT), mean platelet volume (MPV), and platelet distribution width (PDW) with liver fibrosis and hepatic inflammatory activity in nonalcoholic fatty liver disease (NAFLD) and evaluate their noninvasive biomarker potential. This retrospective case-control study included 117 biopsy-confirmed NAFLD patients and 108 healthy controls. NAFLD patients were stratified by METAVIR scoring system fibrosis stage (F0–F4) and inflammatory activity grade (A0–A3). Platelet parameters, biochemical markers, and fibrosis markers were compared. Correlations were assessed using Spearman analysis, and multivariate logistic regression identified independent risk factors. NAFLD patients had lower PLT and higher MPV/PDW levels than controls (all *P* < .05). PLT negatively correlated with fibrosis stage (*r* = −0.640) and inflammatory grade (*r* = −0.556), while MPV (fibrosis: *r* = 0.523; inflammation: *r* = 0.319) and PDW (fibrosis: *r* = 0.417; inflammation: *r* = 0.440) showed positive correlations (all *P* < .01). Decreased PLT, increased MPV, PDW, alanine aminotransferase, aspartate aminotransferase, and procollagen type III N-terminal peptide were independent risk factors for fibrosis progression. Increased PDW, alanine aminotransferase, total cholesterol, hyaluronic acid, and procollagen type III N-terminal peptide were independent risk factors for inflammatory exacerbation (all *P* < .05). Altered platelet parameters (decreased PLT, increased MPV/PDW) in NAFLD patients correlate with, and independently predict, the severity of liver fibrosis and inflammation. These routine hematological indices show promise as noninvasive biomarkers for NAFLD assessment, warranting further prospective validation.

## 1. Introduction

Nonalcoholic fatty liver disease (NAFLD) has emerged as the most prevalent chronic liver disease globally, with its incidence continuously rising, posing a significant challenge to public health worldwide.^[1–3]^ The NAFLD spectrum is broad, ranging from simple hepatic steatosis to nonalcoholic steatohepatitis, the latter being a critical stage leading to liver fibrosis, cirrhosis, and even hepatocellular carcinoma.^[4–7]^ The degree of liver fibrosis and hepatic inflammatory activity are core indicators for assessing the prognosis of NAFLD patients and guiding therapeutic decisions.^[8]^

Currently, liver biopsy remains the gold standard for evaluating liver fibrosis and inflammatory activity.^[9]^ However, its invasive nature, sampling error, risk of complications, high cost, and low patient acceptance limit its widespread application for routine screening and dynamic monitoring of NAFLD.^[10]^ Therefore, there is a significant clinical need and an urgent demand for the development of reliable, convenient, and cost-effective noninvasive biomarkers to accurately assess the status of liver injury in NAFLD.^[11,12]^

Platelet count (PLT) not only play a central role in hemostasis and coagulation but have also been increasingly recognized for their deep involvement in various pathophysiological processes, including inflammation, immune modulation, and tissue repair.^[13]^ The liver is the primary site for thrombopoietin (TPO) synthesis, and impaired liver function can lead to reduced TPO production, thereby affecting PLT counts.^[14]^ In the progression of chronic liver disease, portal hypertension-induced hypersplenism can also accelerate platelet destruction. Mean platelet volume (MPV) and platelet distribution width (PDW) reflect platelet volume heterogeneity and bone marrow production activity; their elevation may be associated with accelerated platelet production or increased destruction related to inflammation and fibrosis.^[15–17]^

Previous studies have explored the relationship between platelet parameters and liver fibrosis in chronic liver diseases, including viral hepatitis and alcoholic liver disease.^[18,19]^ However, research systematically evaluating the association of PLT, MPV, and PDW with biopsy-confirmed liver fibrosis stage (F) and inflammatory activity grade specifically in NAFLD patients, particularly within Chinese populations, remains relatively limited.^[20]^ Clarifying whether these parameters can serve as independent predictors of the severity of liver injury in NAFLD is crucial for their clinical utility.

Therefore, this study, utilizing a retrospective case-control design, aimed to analyze the characteristics of peripheral blood PLT, MPV, and PDW levels in NAFLD patients and to thoroughly investigate their correlations with biopsy-proven liver fibrosis severity and hepatic inflammatory activity, intending to provide new laboratory evidence for the noninvasive assessment of NAFLD.

## 2. Materials and methods

### 2.1. Study population

This retrospective case-control study enrolled 117 patients diagnosed with NAFLD by liver biopsy at the Department of Hepatology, the Fifth People’s Hospital Affiliated to Jiangnan University, between January 2022 and December 2024 (NAFLD group). The diagnosis of NAFLD was based on the criteria outlined in the “Guidelines for the Prevention and Treatment of NAFLD (2024 Updated Edition)”: imaging evidence of hepatic steatosis, and exclusion of excessive alcohol consumption (males > 210 g/wk, females > 140 g/wk), viral hepatitis, drug-induced liver injury, autoimmune liver disease, and genetic metabolic liver diseases. Concurrently, 103 healthy individuals undergoing physical examinations at our hospital’s health examination center were selected as the control group. Inclusion criteria for controls were: no history of liver disease, normal liver function tests, no ultrasonographic evidence of fatty liver or other hepatic lesions, no long-term use of medications known to affect liver function, and no history of excessive alcohol consumption. The NAFLD and control groups were well-matched for age and sex (*P* > .05). This study was approved by the Ethics Committee of the Wuxi Fifth People’s Hospital Affiliated to Jiangnan University, and written informed consent was obtained from all participants.

### 2.2. Histopathological assessment of liver tissue

All 117 NAFLD patients underwent percutaneous liver biopsy guided by ultrasound. Liver tissue specimens were fixed in 10% neutral buffered formalin, paraffin-embedded, sectioned (4–5 μm), and stained with hematoxylin and eosin, Masson’s trichrome, and reticular fiber stain. Two experienced pathologists, blinded to clinical data, independently evaluated the slides according to the METAVIR scoring system. Liver fibrosis stage (F) was defined as: F0, no fibrosis; F1, portal fibrosis without septa; F2, portal fibrosis with few septa; F3, numerous septa with architectural distortion; F4, cirrhosis. Patients were categorized into: no fibrosis group (F0, n = 12), mild fibrosis group (F1–F2, n = 81), and moderate-to-severe fibrosis group (F3–F4, n = 24). Hepatic inflammatory activity grade (A) was defined as: A0, no activity; A1, mild activity; A2, moderate activity; A3, severe activity. Patients were divided into: no inflammation group (A0, n = 2), mild inflammation group (A1, n = 65), and moderate-to-severe inflammation group (A2–A3, n = 50). Disagreements were resolved by joint reevaluation and discussion.

### 2.3. Collection of clinical data and laboratory parameters

General clinical data, including age, sex, height, and weight, were collected. Body mass index (kg/m²) was calculated. Peripheral venous blood samples were collected in the morning after an overnight fast. EDTA-K2 anticoagulated blood was analyzed within 30 minutes for complete blood count parameters, including white blood cell count, PLT, MPV, and PDW, using a BC-7500 automated hematology analyzer (Mindray, China). Serum, separated from clotted blood by centrifugation (3000 rpm for 10 minutes), was used for biochemical analysis on a BK-600 automated biochemical analyzer (Biobase, China), measuring alanine aminotransferase (ALT), aspartate aminotransferase (AST), alkalinephosphatase, γ-glutamyl transferase, fasting blood glucose (Glucose), total cholesterol, triglycerides, low-density lipoprotein cholesterol, high-density lipoprotein cholesterol, and high-sensitivity C-reactive protein. Serum levelsof hyaluronic acid, procollagen type III N-terminal peptide (PCIII-NP), laminin, and type IV collagen were determined by chemiluminescence immunoassay. All procedures strictly followed manufacturers’ instructions.

### 2.4. Statistical analysis

Data analysis was performed using SPSS version 26.0 (IBM Corp., Armonk). After normality testing, normally distributed continuous data were expressed as mean ± standard deviation (*x̄* ± *s*) and compared between 2 groups using the independent samples *t*-test. Non-normally distributed data were expressed as median (interquartile range; *M* [*Q*_1_, *Q*_3_]) and compared using the Mann–Whitney *U* test. Categorical data were expressed as counts (%; n [%]) and compared using the χ² test. Spearman’s rank correlation analysis was used to assess correlations between PLT, MPV, PDW and the stages of liver fibrosis and grades of inflammatory activity. Variables with *P* < .05 in univariate analysis were included in a multivariate binary logistic regression model to identify independent risk factors, calculating odds ratios (OR) and 95% confidence intervals (CI). All tests were 2-sided, and *P* < .05 was considered statistically significant.

## 3. Results

### 3.1. Baseline characteristics and platelet parameters in NAFLD patients and healthy controls

This study included 117 NAFLD patients and 108 healthy controls. Compared to the control group, NAFLD patients exhibited multiple metabolic disorders and abnormal liver function, with significantly elevated levels of ALT, AST, γ-glutamyl transferase, Glucose, low-density lipoprotein, and high-sensitivity C-reactive protein, and significantly decreased high-density lipoprotein levels (all *P* < .05). Regarding platelet parameters, PLT levels were significantly lower in the NAFLD group compared to controls (*P* < .01), whereas MPV and PDW levels were significantly higher (both *P* < .05; Table 1).

**Table 1:** Comparison of general data and laboratory indicators between the NAFLD group and the physical examination group.

	NAFLD group (n = 117)	Control group (n = 103)	*P* value
Age	44.32 ± 9.48	46.21 ± 11.10	.761
Gender: *male/female*	62/55	57/46	.810
BMI	27.45 ± 1.97	24.72 ± 2.13	.417
ALT	86.18 ± 25.01	27.10 ± 17.28	<.01
AST	56.05 ± 19.30	28.35 ± 11.24	<.01
ALP	101.45 ± 38.97	73.81 ± 19.22	.028
GGT	73.62 ± 82.19	44.50 ± 12.17	<.01
Glucose	6.68 ± 2.04	5.84 ± 1.35	<.01
TC	5.78 ± 1.14	5.72 ± 1.49	.22
TG	2.42 ± 1.75	1.77 ± 0.89	.082
LDL	3.60 ± 0.95	2.68 ± 1.10	<.01
HDL	1.22 ± 0.63	1.75 ± 0.48	<.01
WBC (×10^9^/L)	6.14 ± 1.86	6.57 ± 1.70	<.05
PLT (×10^9^/L)	206.09 ± 56.88	228.66 ± 60.24	<.01
MPV	11.23 ± 1.23	10.11 ± 0.95	<.05
PDW	14.03 ± 3.04	11.65 ± 2.07	<.05
hs-CRP	4.15 ± 5.60	3.03 ± 2.94	<.05

ALP = alkaline phosphatase, ALT = alanine aminotransferase, AST = aspartate aminotransferase, BMI = body mass index, GGT = gamma-glutamyl transferase, HDL = high-density lipoprotein, hs-CRP = high-sensitivity C-reactive protein, LDL = low-density lipoprotein, MPV = mean platelet volume, NAFLD = non-alcoholic fatty liver disease, PDW = platelet distribution width, PLT = platelet count, TC = total cholesterol, TG = triglycerides, WBC = white blood cell count.

### 3.2. Association of platelet parameters with the severity of liver fibrosis in NAFLD patients

Among the 117 NAFLD patients, 12 had no fibrosis (F0), 81 had mild fibrosis (F1–F2), and 24 had moderate-to-severe fibrosis (F3–F4). With increasing severity of liver fibrosis, PLT levels progressively decreased (*P* < .01), while MPV and PDW levels showed an increasing trend (both *P* < .05; Table 2). Spearman correlation analysis confirmed that PLT was significantly negatively correlated with F (*r* = −0.640, *P* < .01), whereas MPV (*r* = 0.523, *P* < .01) and PDW (*r* = 0.417, *P* < .01) were significantly positively correlated with F (Table 3). Multivariate logistic regression analysis (Table 4) revealed that after adjusting for other confounding factors, decreased PLT (OR = 0.242, 95% CI: 0.148–0.661, *P* = .020), increased MPV (OR = 1.895, 95% CI: 1.286–2.792, *P* = .011), and increased PDW (OR = 3.832, 95% CI: 1.163–7.850, *P* = .007) were independent risk factors for the progression of liver fibrosis in NAFLD patients. Additionally, ALT, AST, and PCIII-NP were also identified as independent risk factors for fibrosis progression.

**Table 2:** Comparison of general data and laboratory indicators between the mild group and the moderate to severe group.

	Mild group (n = 81)	Moderate to severe group (n = 24)	*P* value
Age	51.34 ± 8.12	55.27 ± 10.09	3.002
Gender: male/female	34/47	4/20	1.877
BMI	26.11 ± 3.05	27.14 ± 3.68	.157
ALT	83.79 ± 13.96	85.87 ± 18.26	<.05
AST	44.70 ± 22.34	54.56 ± 26.34	<.05
ALP	101.93 ± 31.93	93.68 ± 34.27	.103
GGT	109.14 ± 21.01	91.03 ± 18.73	.081
Glucose	5.31 ± 1.25	5.65 ± 1.47	.210
TC	4.70 ± 1.36	4.60 ± 1.05	.190
TG	1.79 ± 0.99	2.09 ± 1.92	<.05
LDL	2.88 ± 0.98	2.90 ± 0.94	<.01
HDL	1.04 ± 0.30	1.03 ± 0.27	<.01
WBC (×10^9^/L)	6.70 ± 1.50	5.61 ± 1.38	<.01
PLT (×10^9^/L)	223.67 ± 93.19	180.08 ± 58.60	<.01
MPV	11.24 ± 1.43	11.66 ± 1.20	<.05
PDW	14.03 ± 3.46	15.01 ± 3.08	<.05
hs-CRP	4.45 ± 1.96	4.86 ± 1.44	.360
Hyaluronic acid	86.05 ± 35.37	122.03 ± 22.28	<.01
Type III procollagen N-terminal peptide	11.47 ± 6.35	15.92 ± 6.94	<.01
Laminin	72.29 ± 27.28	88.95 ± 44.09	<.05
Type IV collagen	55.83 ± 21.14	105.91 ± 41.09	<.05

ALP = alkaline phosphatase, ALT = alanine aminotransferase, AST = aspartate aminotransferase, BMI = body mass index, GGT = gamma-glutamyl transferase, HDL = high-density lipoprotein, hs-CRP = high-sensitivity C-reactive protein, LDL = low-density lipoprotein, MPV = mean platelet volume, PDW = platelet distribution width, PLT = platelet count, TC = total cholesterol, TG = triglycerides, WBC = white blood cell count.

**Table 3:** Analysis of the correlation between PLT, MPV, PDW and the degree of liver fibrosis.

Indicator	*r*	*P*
PLT	−0.640	<.01
MPV	0.523	<.01
PDW	0.417	<.01

MPV = mean platelet volume, PDW = platelet distribution width, PLT = platelet count.

**Table 4:** Analysis of influencing factors of liver fibrosis aggravation in patients with NAFLD.

Influencing factors	β	SE	Wald χ^2^	*P*	OR	95% Cl
PLT	0.236	0.335	6.468	.020	0.242	0.148–0.661
MPV	0.201	0.380	8.004	.011	1.895	1.286–2.792
PDW	1.206	0.197	4.869	.007	3.832	1.163–7.850
WBC	0.107	0.071	12.734	.025	3.967	1.427–7.382
ALT	0.214	0.143	7.205	.008	1.918	1.803–2.194
AST	0.429	0.214	19.837	<.001	6.749	1.156–9.875
TG	0.536	0.286	5.628	.022	3.226	1.988–5.013
HDL	0.643	0.429	17.490	.028	1.579	1.302–1.728
LDL	0.750	0.500	8.376	.059	4.652	1.745–8.660
Hyaluronic acid	0.857	0.714	13.752	1.060	1.508	1.091–3.509
Type III procollagen N-terminal peptide	0.964	0.786	6.319	<.001	3.791	1.619–6.247
Laminin	1.286	0.857	10.582	.624	2.843	1.237–4.081
Type IV collagen	1.179	0.929	15.901	1.044	1.936	1.584–2.303

ALT = alanine aminotransferase, AST = aspartate aminotransferase, HDL = high-density lipoprotein, LDL = low-density lipoprotein, MPV = mean platelet volume, NAFLD = non-alcoholic fatty liver disease, PDW = platelet distribution width, PLT = platelet count, TG = triglycerides, WBC = white blood cell count.

### 3.3. Association of platelet parameters with hepatic inflammatory activity in NAFLD patients

Among NAFLD patients, 2 had no inflammation (A0), 65 had mild inflammation (A1), and 50 had moderate-to-severe inflammation (A2–A3). With increasing grades of inflammatory activity, PLT levels also showed a decreasing trend (*P* < .01), while MPV and PDW levels increased (both *P* < .05; Table 5). Spearman correlation analysis indicated that PLT was significantly negatively correlated with the grade of inflammatory activity (*r* = −0.556, *P* < .01), while MPV (*r* = 0.319, *P* < .01) and PDW (*r* = 0.440, *P* < .01) were significantly positively correlated with the grade of inflammatory activity (Table 6). Multivariate logistic regression analysis (Table 7) demonstrated that increased PDW (OR = 1.748, 95% CI: 1.295–2.361, *P* < .001) was an independent risk factor for the exacerbation of hepatic inflammatory activity in NAFLD patients. Furthermore, ALT, total cholesterol, hyaluronic acid, and PCIII-NP were also identified as independent risk factors for increased inflammatory activity.

**Table 5:** Comparison of general data and laboratory indicators between the mild group and the moderate to severe group.

	Mild group (n = 65)	Moderate to severe group (n = 50)	*P* value
Age	50.49 ± 9.04	53.88 ± 11.30	2.046
Gender: male/female	33/32	10/40	1.877
BMI	25.37 ± 3.80	26.09 ± 4.11	1.570
ALT	85.54 ± 13.20	86.53 ± 12.81	<.05
AST	44.55 ± 27.39	52.46 ± 23.09	.080
ALP	95.36 ± 32.45	108.46 ± 44.28	.051
GGT	93.23 ± 37.35	113.88 ± 24.16	1.306
Glucose	4.87 ± 0.73	5.47 ± 1.30	.900
TC	4.88 ± 1.13	4.98 ± 1.17	<.01
TG	1.87 ± 0.97	2.00 ± 1.25	<.05
LDL	3.28 ± 0.98	3.18 ± 1.11	<.05
HDL	1.05 ± 0.29	1.06 ± 0.33	<.01
WBC (×10^9^/L)	6.56 ± 2.14	5.66 ± 1.50	<.01
PLT (×10^9^/L)	225.14 ± 66.64	193.54 ± 65.60	<.01
MPV	10.98 ± 1.17	11.51 ± 1.18	<.05
PDW	13.54 ± 2.90	14.41 ± 2.81	<.05
hs-CRP	3.87 ± 2.46	3.95 ± 2.75	.285
Hyaluronic acid	67.31 ± 30.21	115.66 ± 23.48	<.05
Type III procollagen N-terminal peptide	11.54 ± 5.29	16.35 ± 6.64	<.01
Laminin	72.87 ± 23.59	87.28 ± 36.76	<.01
Type IV collagen	49.26 ± 24.60	89.90 ± 39.02	.053

ALP = alkaline phosphatase, ALT = alanine aminotransferase, AST = aspartate aminotransferase, BMI = body mass index, GGT = gamma-glutamyl transferase, HDL = high-density lipoprotein, hs-CRP = high-sensitivity C-reactive protein, LDL = low-density lipoprotein, MPV = mean platelet volume, PDW = platelet distribution width, PLT = platelet count, TC = total cholesterol, TG = triglycerides, WBC = white blood cell count.

**Table 6:** Analysis of the correlation between PLT, MPV, PDW and the degree of inflammatory activity in liver tissues.

Indicator	*r*	*P*
PLT	−0.556	<.01
MPV	0.319	<.01
PDW	0.440	<.01

MPV = mean platelet volume, PDW = platelet distribution width, PLT = platelet count.

**Table 7:** Analysis of influencing factors for hepatic inflammatory activity in NAFLD patients.

Influencing factors	β	SE	Wald χ^2^	*P*	OR	95% Cl
PLT	0.318	0.621	15.782	.078	1.137	0.512–1.743
MPV	0.424	0.253	28.301	.058	1.926	1.674–3.902
PDW	1.393	0.807	3.119	<.001	1.748	1.295–2.361
WBC	1.506	0.049	22.507	.090	2.314	1.721–5.438
ALT	0.813	0.093	9.465	.020	4.875	1.183–8.173
TC	0.475	0.116	1.874	<.001	2.654	1.931–4.679
TG	0.769	0.309	11.993	.071	5.105	1.348–9.204
HDL	1.338	0.584	25.036	.228	4.589	1.067–7.119
LDL	0.692	0.460	5.102	.090	6.741	1.759–11.382
Hyaluronic acid	1.250	0.690	18.640	<.001	3.382	1.226–6.918
Type III procollagen N-terminal peptide	0.588	0.172	7.759	<.001	1.987	1.849–3.007
Laminin	1.054	0.208	21.088	.408	1.279	0.093–1.508

ALT = alanine aminotransferase, HDL = high-density lipoprotein, LDL = low-density lipoprotein, MPV = mean platelet volume, NAFLD = non-alcoholic fatty liver disease, PDW = platelet distribution width, PLT = platelet count, TC = total cholesterol, TG = triglycerides, WBC = white blood cell count.

## 4. Discussion

This study aimed to evaluate the association of platelet parameters (PLT, MPV, PDW) with liver biopsy-confirmed fibrosis and inflammatory activity in patients with NAFLD. Our principal findings indicate that, compared to healthy controls, NAFLD patients exhibited significantly decreased PLT and significantly elevated MPV and PDW. More importantly, these alterations were closely correlated with the severity of liver fibrosis and inflammatory activity. After adjusting for confounding factors, decreased PLT, increased MPV, and increased PDW remained independent predictors of advanced liver fibrosis, and increased PDW was an independent predictor of heightened inflammatory activity.

The decrease in PLT among NAFLD patients aligns with most previous studies^[21]^ and can be attributed to reduced hepatic TPO synthesis, portal hypertension-induced hypersplenism, and platelet consumption in the chronic inflammatory state.^[22–24]^ The elevation in MPV and PDW reflects an accelerated release of larger, newly formed platelets from the bone marrow to meet increased demand, suggesting altered platelet turnover kinetics in the pathological process of NAFLD, potentially linked to inflammation and tissue repair processes.^[25,26]^

The core contribution of this study lies in demonstrating that these 3 routine platelet parameters are not only associated with the pathological severity of NAFLD but also possess independent predictive value. This resonates with the rationale behind established noninvasive liver fibrosis scores that incorporate PLT (e.g., Aspartate aminotransferase to platelet ratio index, fibrosis-4 index) and suggests that MPV and PDW may also provide additional information for noninvasive assessment.^[27]^ Our findings provide a basis for developing or optimizing noninvasive diagnostic models for NAFLD, indicating that these readily available and inexpensive markers hold promise for aiding clinical judgment of liver injury severity.

Although this study included a relatively substantial sample size and was based on the gold standard of liver biopsy, certain limitations exist. As a single-center retrospective study, the generalizability of the results may be limited, and dynamic observations were not performed. Furthermore, while multiple confounders were adjusted for, unmeasured factors might still have an impact.^[28,29]^

In conclusion, abnormal changes in PLT, MPV, and PDW serve as important signals of hepatic pathological progression in NAFLD patients. These platelet parameters, as potential noninvasive biomarkers, show promise in assisting the assessment of liver fibrosis and inflammatory activity in NAFLD. Future research should focus on validating these findings in prospective, multicenter cohorts and exploring their specific utility in integrated diagnostic models to improve risk stratification and clinical management of NAFLD patients.

## 5. Conclusion

The findings of this study demonstrate that patients with NAFLD exhibit significant abnormalities in platelet parameters, primarily characterized by decreased PLT and elevated MPV and PDW. These alterations are closely associated with the severity of liver fibrosis and hepatic inflammatory activity, as confirmed by liver biopsy. Furthermore, multivariate analysis confirmed that decreased PLT, increased MPV, and increased PDW are independent predictors of advanced liver fibrosis or exacerbated inflammatory activity in NAFLD patients. Therefore, PLT, MPV, and PDW, as routine, inexpensive, and readily available hematological indices, hold promise as potential noninvasive biomarkers to aid in assessing the severity and progression of NAFLD. They may assist clinicians in risk stratification and the formulation of individualized management strategies. Nevertheless, their definitive clinical utility requires further validation and optimization through larger-scale, rigorously designed, prospective multicenter studies.

## Author contributions

**Conceptualization:** Zefeng Zhang.

**Data curation:** Zefeng Zhang.

**Formal analysis:** Zefeng Zhang.

**Software:** Zefeng Zhang.

**Supervision:** Jun Wang.

**Validation:** Jun Wang.

**Writing** – **original draft:** Jun Wang.

**Writing** – **review & editing:** Jun Wang.
